# Cannabinoid receptor subtype 2 (CB_2_R) agonist, GW405833 reduces agonist-induced Ca^2+^ oscillations in mouse pancreatic acinar cells

**DOI:** 10.1038/srep29757

**Published:** 2016-07-19

**Authors:** Zebing Huang, Haiyan Wang, Jingke Wang, Mengqin Zhao, Nana Sun, Fangfang Sun, Jianxin Shen, Haiying Zhang, Kunkun Xia, Dejie Chen, Ming Gao, Ronald P. Hammer, Qingrong Liu, Zhengxiong Xi, Xuegong Fan, Jie Wu

**Affiliations:** 1 Department of Infectious Diseases, Xiangya Hospital, Central South University, and Key Laboratory of Viral Hepatitis, Hunan Province, Changsha 410008, China; 2 Departments of Neurology and Neurobiology, Barrow Neurological Institute, St. Joseph’s Hospital and Medical Center, Phoenix AZ 85013, USA; 3 Department of Physiology, Shantou University Medical College, Shantou, Guangdong 515041, China; 4 Intramural Research Program, National Institute on Drug Abuse, Baltimore, MD 21224, USA; 5 Department of Basic Medical Sciences, University of Arizona College of Medicine, Phoenix, AZ 85004, USA; 6 Departments of Pharmacology and Psychiatry University of Arizona College of Medicine Tucson, AZ, 85721, USA.

## Abstract

Emerging evidence demonstrates that the blockade of intracellular Ca^2+^ signals may protect pancreatic acinar cells against Ca^2+^ overload, intracellular protease activation, and necrosis. The activation of cannabinoid receptor subtype 2 (CB_2_R) prevents acinar cell pathogenesis in animal models of acute pancreatitis. However, whether CB_2_Rs modulate intracellular Ca^2+^ signals in pancreatic acinar cells is largely unknown. We evaluated the roles of CB_2_R agonist, GW405833 (GW) in agonist-induced Ca^2+^ oscillations in pancreatic acinar cells using multiple experimental approaches with acute dissociated pancreatic acinar cells prepared from wild type, CB_1_R-knockout (KO), and CB_2_R-KO mice. Immunohistochemical labeling revealed that CB_2_R protein was expressed in mouse pancreatic acinar cells. Electrophysiological experiments showed that activation of CB_2_Rs by GW reduced acetylcholine (ACh)-, but not cholecystokinin (CCK)-induced Ca^2+^ oscillations in a concentration-dependent manner; this inhibition was prevented by a selective CB_2_R antagonist, AM630, or was absent in CB_2_R-KO but not CB_1_R-KO mice. In addition, GW eliminated L-arginine-induced enhancement of Ca^2+^ oscillations, pancreatic amylase, and pulmonary myeloperoxidase. Collectively, we provide novel evidence that activation of CB_2_Rs eliminates ACh-induced Ca^2+^ oscillations and L-arginine-induced enhancement of Ca^2+^ signaling in mouse pancreatic acinar cells, which suggests a potential cellular mechanism of CB_2_R-mediated protection in acute pancreatitis.

Acute pancreatitis is an inflammatory disease, which has several causes and symptoms and requires immediate medical attention^1^,^2^. In clinical practice, there are still no efficient drugs that specifically treat acute pancreatitis^1^. Emerging evidence demonstrates that a primary event initiating the process of acute pancreatitis is the excessive release of Ca^2+^ from intracellular stores^3^. These studies provide a promising therapeutic strategy—the blockade of Ca^2+^ release-activated Ca^2+^ currents in pancreatic acinar cells may provide significant protection against Ca^2+^ overload, intracellular protease activation, and necrosis, which are the major triggers of acute pancreatitis.

The cannabinoid receptor type 2 (CB_2_R) is a G protein-coupled receptor that, in humans, is encoded by the *CNR2* gene^4^. CB_2_Rs are predominantly expressed in the periphery, especially in immune cells, suggesting that CB_2_R mediates the effects of cannabinoids mainly in the immune system. For example, activation of CB_2_Rs inhibits adenylyl cyclase via Gi/Go_α_ subunits and causes a reduction in the intracellular level of cyclic adenosine monophosphate (cAMP)^5^,^6^, which has been implicated in a variety of modulatory functions including immune suppression, induction of apoptosis, and induction of cell migration^7^. Thus, CB_2_R agonists may be useful candidates for treating inflammatory diseases and pain^8^. Consistent with these findings, increased CB_2_R expression has been observed in spinal cord, dorsal root ganglion, and activated microglia in a rodent model of neuropathic pain, as well as in human hepatocellular carcinoma tumor samples^9^. In addition, emerging data demonstrate that CB_2_R mRNA and protein are expressed in pancreatic acinar cells, and activation of these CB_2_Rs prevents acinar cell pathogenesis in an animal model of pancreatitis^10^. However, whether the activation of CB_2_R modulates intracellular Ca^2+^ signals in pancreatic acinar cells is largely unknown. Specifically, it is unknown whether an agent that induces pancreatitis (e.g., L-arginine) enhances Ca^2+^ oscillations and whether application of a CB_2_R agonist eliminates L-arginine-induced enhancement of Ca^2+^ oscillations in pancreatic acinar cells.

In this study, we address these important questions using patch-clamp and confocal Ca^2+^ imaging approaches combined with immunohistochemistry using wild-type (WT), CB_1_R-knockout (KO), and CB_2_R-KO mice.

## Methods

All experimental protocols were approved by and performed in accordance with guidelines set by the animal care and use and ethical committees at the Barrow Neurological Institute, Xiangya Hospital (Hunan, Changsha, China), and Shantou University Medical College (Shantou, Guangdong, China).

### Animals

Mice used for this study were adult (4–6 month old), male, CD1 mice (Charles River Laboratories International, Inc., Wilmington, MA, USA). In addition, WT, CB_1_RKO^11^, and CB_2_RKO mice^12^ with C57BL/6J genetic backgrounds were initially provided by Dr. Zheng-Xiong Xi at the National Institute on Drug Abuse (NIDA; Bethesda, MD, USA), and were then bred in animal facilities at the Barrow Neurological Institute, which are accredited by the Association for Assessment and Accreditation of Laboratory Animal Care. Genotyping was performed at the NIDA Intramural Research Program before experiments were begun. All animals used in the experiments were matched for age (8–14 weeks) and weight (25–35 grams).

### Mouse Pancreatic Acinar Cell Preparation

Acute isolated pancreatic cells were prepared as previously described^13^,^14^,^15^. In brief, pancreatic glands were taken from isoflurane-anesthetized mice, and fragments of the tissue were minced and digested using collagenase (200 U/mL, 25–30 min, 37 °C; Wako Pure Chemicals, Osaka, Japan) in the presence of 1-mM Ca^2+^. After collagenase digestion, the cell suspension was gently pipetted to obtain further separation of the cells, and then washed with physiological saline. A 100-μL volume of cell suspension was then poured into extracellular solution in a 2-mL experimental bath. The isolated cells usually adhered to the bottom within 15–20 min and were used for recording within 3 h after preparation. All experiments were performed at room temperature (22 ± 1 °C).

### Whole-Cell Patch-Clamp Recording and Perforated-Patch Recording

Conventional whole-cell patch-clamp recording was used to record the Ca^2+^-activated Cl^−^ currents for monitoring intracellular Ca^2+^ signal oscillations, as reported previously^13^,^14^. The recording pipettes, made from borosilicate glass capillaries, had a resistance of 3–5 MΩ when filled with pipette solution. After a GΩ seal was established between the cell membrane and the pipette, a whole-cell configuration was achieved by brief negative suction. Transmembrane currents were recorded with a patch-clamp amplifier (Axopatch 200B; Molecular Devices, Sunnyvale, CA, USA) at a holding potential (V_H_) of −30 mV. For perforated-patch recording, amphotericin B (200 μg/mL) was dissolved into the pipette solution. In these studies, we did not compensate for series resistance.

### Drug Application

A stream of standard extracellular solution was continuously perfused over the cell during recording. A computer-controlled U-tube system was used for drug application^16^. For intracellular drug application, the drug was added into pipette solution, and establishment of a whole-cell configuration allowed the drug to diffuse into the recorded cell.

### Amylase Estimation

Serum amylase activity was measured using the AMS assay kit (Nanjing Jiancheng Corp., Nanjing, China) and a microplate reader, following the manufacturer’s recommendations.

### Myeloperoxidase Estimation

To measure myeloperoxidase (MPO) activity, lung tissues were immediately homogenized on ice in 10 volumes of normal saline. MPO activity was measured using the MPO assay kit (Nanjing Jiancheng Corp., Nanjing, China) and a microplate reader, following the manufacturer’s recommendations.

### CB_2_R Immunoblot Assay

WT, CB_1_R-KO, and CB_2_R- KO mice (3 mice for each group) were anesthetized and quickly perfused with saline to flash all blood cells. Both whole striatum and spleen tissue were dissected out, snap frozen, and kept on dry ice. All the tissues were homogenized in cell lysis buffer (Cell Signaling Technology, Inc., Danvers, MA, USA) using a sonicator and centrifuge at 15,000 rpm for 15 min at 4 °C to get supernatant. The protein concentration for each sample was quantified with a Bio-Rad Protein Assay (Bio-Rad Laboratories, Hercules, CA, USA). A total of 20-μg protein (spleen) or 40-μg protein (striatum) were loaded and separated by SDS-PAGE in a 4–15% gradient gel for the detection of endogenous calnexin (Enzo Life Sciences, SPA865) and CB_2_R (NIDA-5633) by using Invitrogen blotting and transferring modules (Grand Island, NY, USA). Membranes were blocked for 2 h at room temperature with Licor Odyssey blocking buffer (LI-COR Biosciences, Lincoln, NE, USA) after washing 3 times with phosphate-buffered saline containing 0.1% Tween-20. Membranes were first incubated with either anti-CB_2_ (1:500 NIDA-5633 Ab) or anti-calnexin (1:1,000) antibody overnight at 4 °C. After washing 3 times, the membranes were incubated with goat anti-rabbit IgG (IRDye 680CW) (1:2,500) for 1.5 h at room temperature. Then the membranes were washed 3 times and then scanned in a Licor Odyssey Sa Imaging System (LI-COR Biosciences).

### Immunohistochemistry

Sections were first blocked in 5% bovine serum albumin (BSA) and 0.5% Triton X-100 in phosphate buffer (PB) for 2 h at room temperature. Then, sections were incubated with 1:500 NIDA-5633 mCB_2_R antibody (Genemed Synthesis Inc, San Antonio, TX, USA) at 4 °C overnight. After washing 3 times with 0.1 M PB, sections were incubated with Alexa Fluor 488 goat anti-rabbit secondary antibody (Invitrogen, Carlsbad, CA, USA) in 5% BSA and 0.5% Triton X-100 PB for 2 h at room temperature. Sections were then washed, mounted, and cover slipped. Images were taken with a fluorescence microscope (Nikon Eclipse 80i) equipped with a digital camera (Nikon Instruments Inc., Melville, NY, USA).

### Confocal Ca^2+^ Imaging

Dissociated pancreatic acinar cells were first incubated with fluo-4-AM (15 μM) (Molecular Probes, Eugene, OR, USA) for 15 min, followed by a 10-min rest allowing for de-esterification of the indicator. Confocal imaging was performed using an Olympus FluoView FV1000 microscope (Olympus Corporation, Center Valley, PA, USA) equipped with an argon laser (488 nm) and a UPLSAPO 40×, 0.95 NA objective. X-Y imaging was performed at a rate of 1.644 s per frame, 400 frames total, with a resolution of 512 × 512. Fluorescent fluo-4 signal was measured using ImageJ v.1.47 (available from the U.S. National Institutes of Health, Bethesda, MD, USA; http://imagej.nih.gov/ij/).

### Solution and Chemicals

Standard extracellular solution contained (in mM): 140 NaCl, 1.0 CaCl_2_, 4.7 KCl, 1.13 MgCl_2_, 10 glucose, and 10 HEPES, adjusted to pH 7.2 with NaOH. Pipette solution contained (in mM) 140 KCl, 1.13 MgCl_2_, 5 Na_2_ATP, 0.24 EGTA, 10 glucose and 10 HEPES, pH 7.2. Drugs used in this study were GW405833 (Supplemental Fig. 1), JWH133, ACEA, and AM630, cholecystokinin (CCK), which were purchased from Tocris Bioscience (Minneapolis, MN, USA). Acetylcholine (ACh), amphotericin B, and L-arginine were purchased from Sigma-Aldrich (St. Louis, MO, USA).

CB_2_R antibodies, NIDA-5633 mCB2-Ab (customer-designed, raised in rabbit) that recognize the C-terminal (326-340 aa) of mCB_2_Rs, were produced by Genemed Synthesis Inc. (San Antonio, TX, USA).

### Statistics

For patch-clamp experiments, the Ca^2+^-activated Cl^−^ current responses were presented as the current charge (current area/Cm/min), and then the drug-induced changes were compared to the baseline level of charge (induced by ACh). When data were obtained from the same recorded cell and the changes of ACh response were compared before, during, and after testing drug exposure, a paired Student *t* test was used. To compare the effect of the tested drug between 2 groups of animals (e.g., saline group and L-arginine group), the unpaired Student *t* test was used. To analyze multiple effects, one-way analysis of variance (ANOVA) with Tukey’s post hoc tests were used.

## Results

### CB_2_Rs Are Expressed on Mouse Pancreatic Acinar Cells

**Figure 1:**
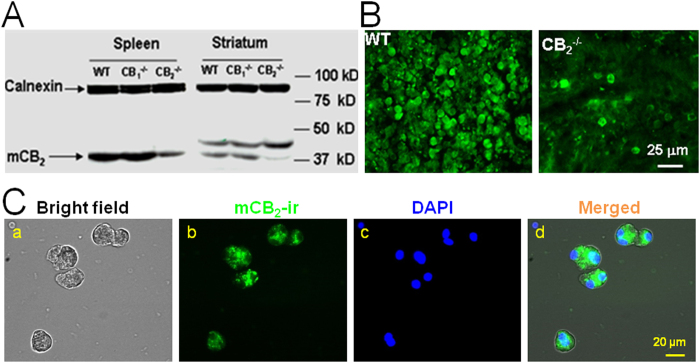
Identification of CB_2_R expression in mouse pancreatic acinar cells. (**A**) Western blot assay shows that a high-density CB_2_-immunoreactive band is detected in both spleen and striatal tissues in WT and CB_1_-KO mice, but is undetectable in striatal tissues or substantially reduced in CB_2_-rich spleen tissues in CB_2_-KO mice. (**B**) Immunohistochemical assays show high densities of CB_2_-immunostaining in spleen slices of WT mice, which are undetectable or substantially diminished in CB_2_-KO mice. (**C**) Immunocytochemical assays use mouse CB_2_R antibody (NIDA-5633). The bright field photograph (Ca) shows freshly dissociated pancreatic acinar cells. CB_2_-immunostaining (mCB_2_-ir) in single dissociated pancreatic acinar cells illustrates the high densities of CB_2_R proteins (Cb). DAPI staining demonstrates cell nucleus (Cc). The “Merged” image shows superimposed mCB2-ir and DAPI images (Cd).

Under the acutely dissociated acinar cell protocol, the isolated cells exhibited a typical kidney shape with secretion granules in the central area of the cells (Supplemental Fig. 2), suggesting the purity of the acinar cells as previously reported^13^,^15^,^17^. Figure 1A shows the results of the immunoblot assays, illustrating that a CB_2_-positive band was detected (at ~40 kD) in both the spleen and striatal tissues of WT and CB_1_-KO (CB_1_^−/−^), while the densities of this band in CB_2_-KO mice (CB_2_^−/−^) were substantially reduced in CB_2_-rich spleen tissues and almost undetectable in striatal tissues. Figure 1B shows CB_2_R immunostaining with the CB_2_R antibody (NIDA-5633), illustrating that the high densities of CB_2_R immunostaining were detected in the majority of spleen cells of WT mice. In contrast, a very low density of CB_2_-like staining was detected in a minority of spleen cells in CB_2_R KO mice, suggesting that the NIDA-5633 antibody used is highly mouse CB_2_R-specific. We then used this antibody to detect CB_2_R expression in single isolated acinar cells. Figure 1C demonstrates the photographs taken in bright field (Ca), mouse CB_2_R antibody (mCB_2_-ir, Cb), DAPI (Cc), and merged mCB_2_-ir and DAPI (Cd). We found high densities of CB_2_R immunolabeling in pancreatic acinar cells (Fig. 1Cb,d). These results suggest that CB_2_R protein is expressed in dissociated mouse pancreatic acinar cells.

### Effects of GW405833 on ACh-Induced Ca^2+^ Oscillations

**Figure 2:**
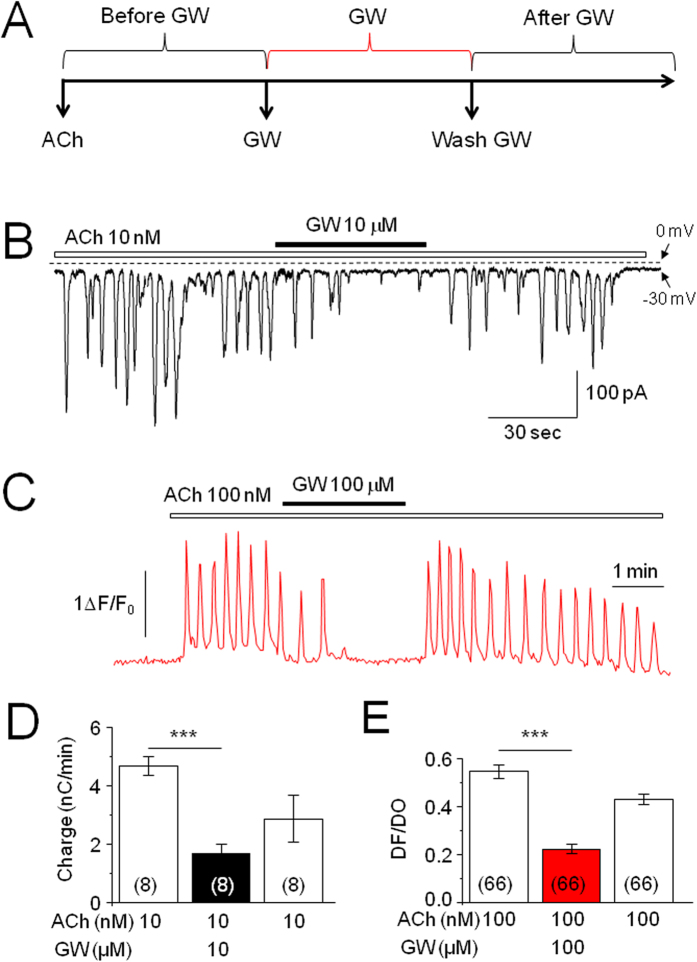
Effects of the CB_2_R agonist on ACh-induced Ca^2+^ oscillations in dissociated pancreatic acinar cells. (**A**) Experimental protocol shows continuous exposure to ACh (baseline), addition of GW on top of ACh, and washout of GW (with ACh). A typical trace of ACh-induced Ca^2+^ oscillations measured using patch-clamp whole-cell recording in voltage-clamp mode (measuring Ca^2+^-dependent Cl^−^ current). In the continuous presence of ACh (10 nM), addition of GW (10 μM) reversibly reduced Ca^2+^ oscillations. (**B**) A typical trace of ACh-induced Ca^2+^ oscillations measured using confocal Ca^2+^ imaging; GW (100 μM) inhibited ACh (100 nM)-induced Ca^2+^ oscillations. Statistical analysis shows that GW significantly reduces ACh-induced Ca^2+^ oscillations in both patch-clamp recording (**C**) and Ca^2+^ imaging experiments (**D**). (**D**) The net charge of ACh-induced baseline Ca^2+^ oscillations (prior to GW application) is compared to the charge during GW application (+GW) and during washout of GW (Washout). Numbers in parentheses indicate the number of cells tested. Columns indicate the mean of current charge ± SEM (left) and the mean DF/DO ± SEM (right) as compared to the baseline level. ***Indicates *p* < 0.001 for the value compared to baseline level. Statistic comparison between the levels of baseline and washout of GW showed significance (*p* < 0.05) in patch-clamp data (Fig. 2D left panel) and in Ca^2+^ imaging data (*p* < 0.01, Fig. 2D right panel).

In acutely dissociated pancreatic acinar cells, low nanomolar concentrations of ACh induced intracellular Ca^2+^ signal oscillations, which can be detected using patch-clamp recording and Ca^2+^ imaging as previously reported^13^,^14^,^18^,^19^,^20^. Our initial series of experiments was designed to test the effects of the CB_2_R agonist, GW405833 (GW), on ACh-induced Ca^2+^ oscillations. Figure 2A demonstrates an experimental protocol, in which the ACh (e.g., 10 nM) is continuously perfused to the recorded cell through a bath (U-tube) to get Ca^2+^ oscillation response (as a baseline). Then, the GW is added to the bath perfusion in the presence of ACh. Finally, the GW is washed out with the same concentration of ACh. With this protocol, the ACh is continuously perfused throughout the recording period, and we can compare the change of ACh-induced Ca^2+^ oscillations before GW perfusion (baseline), during GW perfusion, and after GW washout in the same recorded cell. For statistical analysis of the effects of GW on ACh-induced Ca^2+^ oscillations, we measured baseline oscillations as the charge (current area/Cm/min)^18^ and compared the changes of Ca^2+^ oscillations during GW perfusion and after washout of GW to the baseline. Our data showed that in the continuous presence of 10 nM ACh, 10 μM GW reduced Ca^2+^ oscillations, and this inhibitory effect was reversed after washout (Fig. 2B). A similar inhibitory effect by GW (100 μM) was also observed on 100 nM ACh-induced Ca^2+^ oscillations using confocal Ca^2+^ imaging (Fig. 2C). Statistical analysis of the Ca^2+^ oscillation signal from 8 cells tested showed that GW significantly reduced ACh-induced Ca^2+^ oscillations from baseline level of −4.69 ± 0.32 to −1.68 ± 0.32 nC/min (the level after GW exposure, n = 8, paired *t* test *p* < 0.001, Fig. 2D). Ca^2+^ imaging experiments also showed a similar inhibition of Ca^2+^ oscillations by GW (n = 66, paired *t* test *p* < 0.001, Fig. 2E). After washout of GW, Ca^2+^ oscillations were partially recovered in both patch recording and Ca^2+^ imaging. These results suggest that activation of CB_2_R by GW inhibits ACh-induced intracellular Ca^2+^ signals in freshly isolated pancreatic acinar cells.

### GW Inhibits ACh-Induced Ca^2+^Oscillations in a Concentration-Dependent Manner

**Figure 3:**
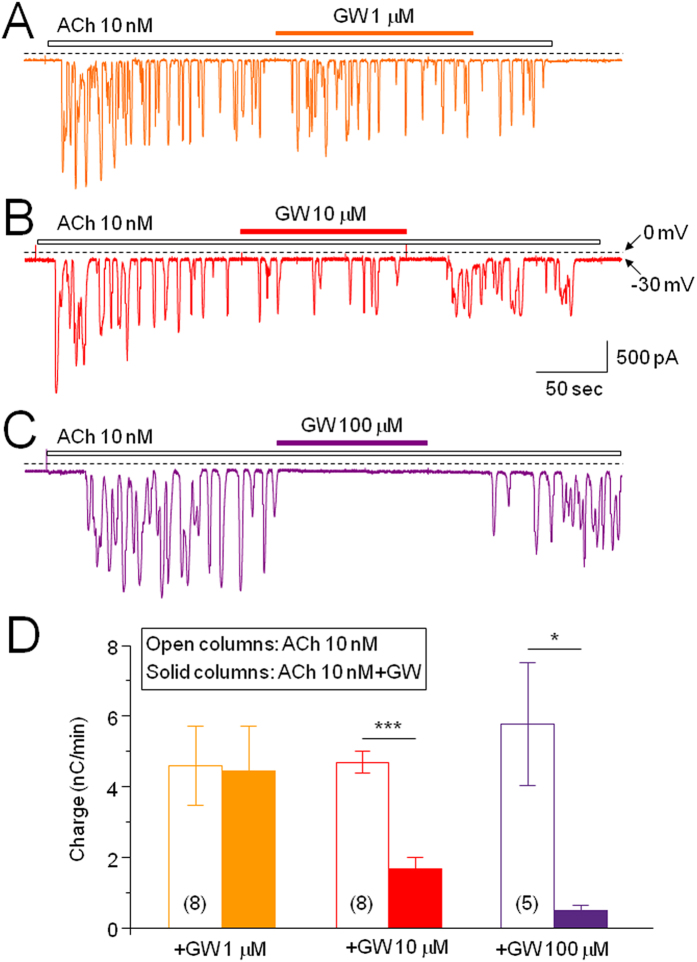
GW inhibits ACh (10 nM)-induced Ca^2+^ oscillations in a concentration-dependent manner. Typical traces show the effect of different concentrations of GW: (**A**) 1 μM, (**B**) 10 μM, (**C**) 100 μM. (**D**) Bar graph summarizes the concentration-dependent effect of GW on ACh-induced Ca^2+^ oscillations. The number of cells tested is stated for each condition in parentheses. Columns show the mean of charge ± SEM. *Indicates *p* < 0.05, ***Indicates *p* < 0.001 for the values between baseline level of ACh response indicated as open columns at left and the level after GW exposure (solid columns). No asterisk mark means (GW 1 μM group) *p* > 0.05.

To profile the pharmacological effect of GW on ACh-induced Ca^2+^ oscillations, we examined the effects of different concentrations of GW on 10 nM ACh-induced Ca^2+^ oscillations. Figure 3A–C show that GW inhibited Ca^2+^ oscillations in a concentration-dependent manner. In 1 μm GW group, Ca^2+^ oscillation levels were slightly reduced from baseline −4.59 ± 1.11 to −4.46 ± 1.25 nC/min (*p* > 0.05, n = 8). In 10 μm GW group, Ca^2+^ oscillation levels were reduced from baseline −4.69 ± 0.32 to −1.68 ± 0.32 nC/min (*p* < 0.0001, n = 8). In 100 μm GW group, Ca^2+^ oscillation levels were reduced from baseline −5.77 ± 1.75 to −0.50 ± 0.15 nC/min (*p* < 0.05, n = 5). Further comparisons determined that Ca^2+^ oscillation levels differed significantly between the following groups: GW 1 μM vs. 10 μM (*p* < 0.05), GW 1 μM vs. 100 μM (*p* < 0.05), and GW 10 μM vs. 100 μM (*p* < 0.01), which confirms that GW inhibition occurs in a concentration-dependent manner.

### GW Inhibits ACh-Induced Ca^2+^ Oscillations by a Selective Action on CB_2_Rs

**Figure 4:**
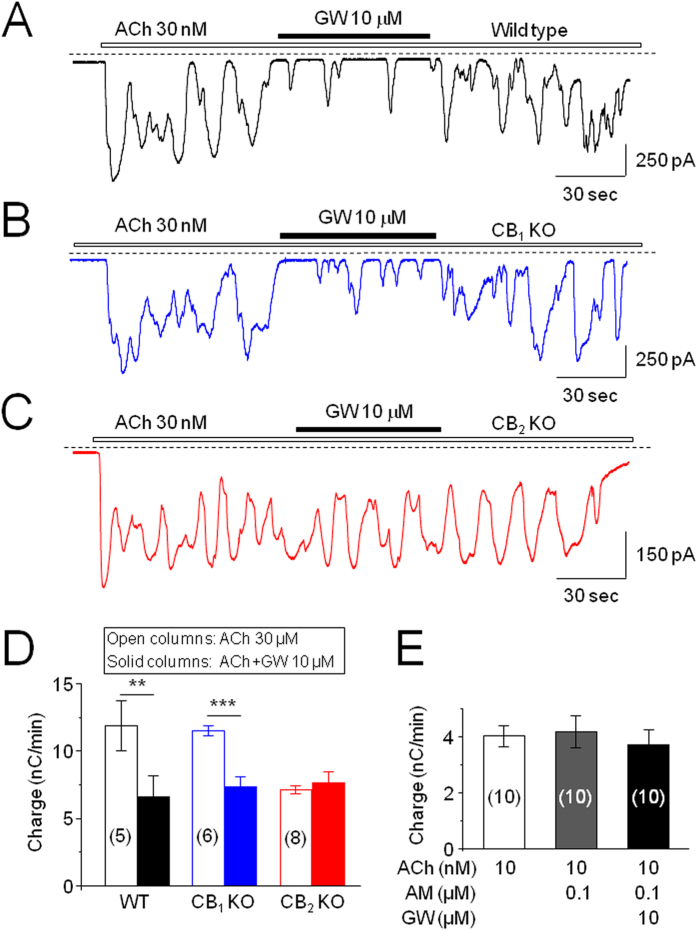
GW (10 μM) inhibits ACh-induced Ca^2+^ oscillations through CB_2_Rs. (**A**) A typical trace shows the effect of GW on ACh (30 nM)-induced Ca^2+^ oscillations in WT mice cells. (**B**) GW fails to inhibit ACh-induced Ca^2+^ oscillations in acinar cells prepared from CB_1_R-KO mice. (**C**) GW inhibits ACh-induced Ca^2+^ oscillations in acinar cells prepared from CB_2_R-KO mice. (**D**) Columns show the mean of charge ± SEM, summarizing the effect of GW on ACh-induced Ca^2+^ oscillations in WT, CB_1_R-KO and CB_2_R-KO mice cells. The number of cells tested is stated for each condition in parentheses. **Indicates *p* < 0.01 compared to the baseline level of ACh response (open columns) to the level after GW exposure (solid columns). (**E**) Bar graph demonstrates that the CB_2_R antagonist (AM630) alone does not significantly affect ACh-induced Ca^2+^ oscillation response (baseline vs. AM630: *p* > 0.05) but abolishes GW-induced inhibition (baseline vs. Am630 + GW: *p* > 0.05).

To address the question of whether GW inhibition of ACh-induced Ca^2+^ oscillations is mediated through CB_2_Rs, we designed three sets of experiments. 1) We tested the effect of a selective CB_2_R antagonist (AM630) on GW inhibition of Ca^2+^ oscillations. 2) We examined GW inhibitory effects on pancreatic acinar cells prepared from CB_1_-KO and CB_2_-KO mice. 3) We evaluated the effects of a selective CB_1_R agonist (ACEA) on ACh-induced Ca^2+^ oscillations. The results of these experiments demonstrated that GW inhibition of ACh-induced Ca^2+^ oscillations was presented in WT (Fig. 4A) and CB_1_R-KO mice (Fig. 4B), but was absent in CB_2_R-KO mice (Fig. 4C). Figure 4D summarizes pooled data demonstrating the effect of GW on 30 nM ACh-induced Ca^2+^ oscillations in WT (*p* < 0.01, n = 5), CB_1_R-KO (*p* < 0.001, n = 6), and CB_2_R-KO (*p* > 0.05, n = 8) mice. Furthermore, co-application of AM630 (0.1 μM) and GW (10 μM) abolished the inhibitory effect of GW on 10 nM ACh-induced Ca^2+^ oscillations (baseline vs. AM630 + GW *p* > 0.05, n = 10), while AM630 alone had no affect (baseline vs. AM630, *p* > 0.05, n = 10, Fig. 4E). Finally, we found that CB_1_R agonist, ACEA (10 μM) also reduced ACh-induced Ca^2+^ oscillations but this effect was likely mediated through ethanol that was used to dissolve ACEA (Supplemental Fig. 3). Together, these results suggest that GW inhibits ACh-induced intracellular Ca^2+^ signaling through the action of CB_2_Rs.

### GW Inhibits ACh-Induced Ca^2+^ Oscillations through Membrane CB_2_Rs

**Figure 5:**
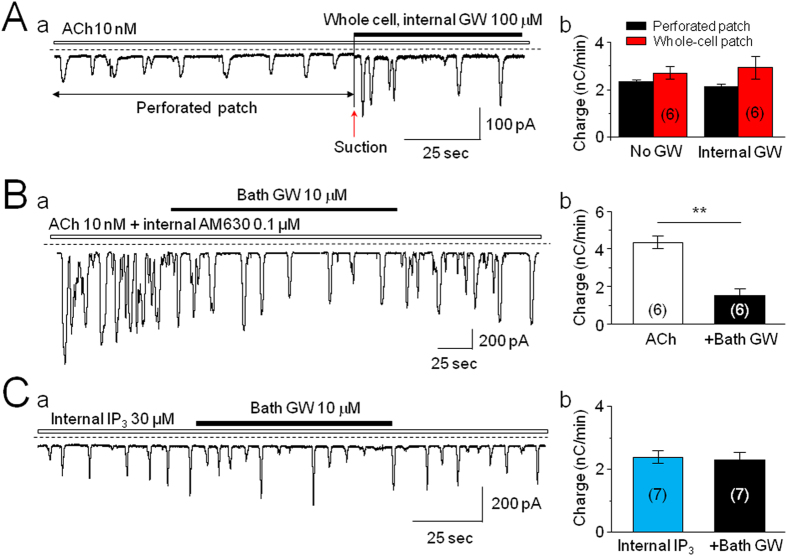
GW inhibits ACh-induced Ca^2+^ oscillations through cytoplasmic, rather than intracellular, CB_2_Rs. (**A**) A typical trace shows ACh-induced Ca^2+^ oscillations between perforated and conventional whole-cell recordings, in which the pipette solution contained 100 μM GW. Infusion of GW into the recorded cell does not reduce ACh-induced Ca^2+^ oscillations (**Aa**). Ab: Summary of pooled data, demonstrating no significant difference between ACh responses with and without intracellular GW (*p* > 0.05, n = 6). (**B**) Internal infusion of AM630 fails to prevent bath-applied GW inhibition of ACh-induced Ca^2+^ oscillations. **Indicates *p* < 0.01. (**C**) Intracellular applied IP_3_-induced Ca^2+^ oscillations are not sensitive to bath-applied GW (Ca). (Cb) Bar graph summarizes the effect of GW on IP_3_-induced Ca^2+^ oscillations and showing no significance before and after GW exposure (*p* > 0.05, n = 7). The number of cells tested is stated for each condition in parentheses.

Our data clearly demonstrated that GW inhibited ACh-induced intracellular Ca^2+^ oscillations. However, it remained unclear whether GW inhibition was mediated through extracellular or intracellular CB_2_Rs. GW could act on extracellular membrane CB_2_Rs and/or modulate muscarinic receptors, or GW could affect intracellular CB_2_Rs, and then modulate signal molecules such as G-protein and/or inositol 1,4,5-trisphosphate (IP_3_) receptors^21^. To distinguish among these possibilities, we designed two experiments, in which, either the CB_2_R agonist (GW) or antagonist (AM630) was applied internally or in which, IP_3_ was applied internally. When GW (100 μM) was added into the recording electrode and a perforated whole-cell recording (amphotericin B) was performed, bath-application of 10 nM ACh induced Ca^2+^ oscillations. When the recording mode was switched from perforated to conventional whole-cell recording by a brief suction, GW was infused into the recorded cell, and no detectable inhibitory effect on ACh-induced Ca^2+^ oscillations was present (Fig. 5A,D). Using the same experimental protocol, we applied AM630 (1 μM) intracellularly and found that internal AM630 failed to prevent bath-applied GW-induced inhibition in the ACh-induced Ca^2+^ oscillations (Fig. 5B,D). In the presence of intracellularly applied IP_3_ (30 μM), which causes IP_3_-induced Ca^2+^ oscillations, GW produced little inhibitory effect on the IP_3_-induced Ca^2+^ oscillations (Fig. 5C,D). These data suggest that GW inhibition of ACh-induced Ca^2+^ oscillations is not mediated through intracellular IP_3_ receptors. Together, these results suggest that GW inhibition of intracellular Ca^2+^ oscillations is mediated through CB_2_Rs on the surface of the cytoplasmic membrane.

### Effects of GW on CCK-Induced Ca^2+^ Oscillations

**Figure 6:**
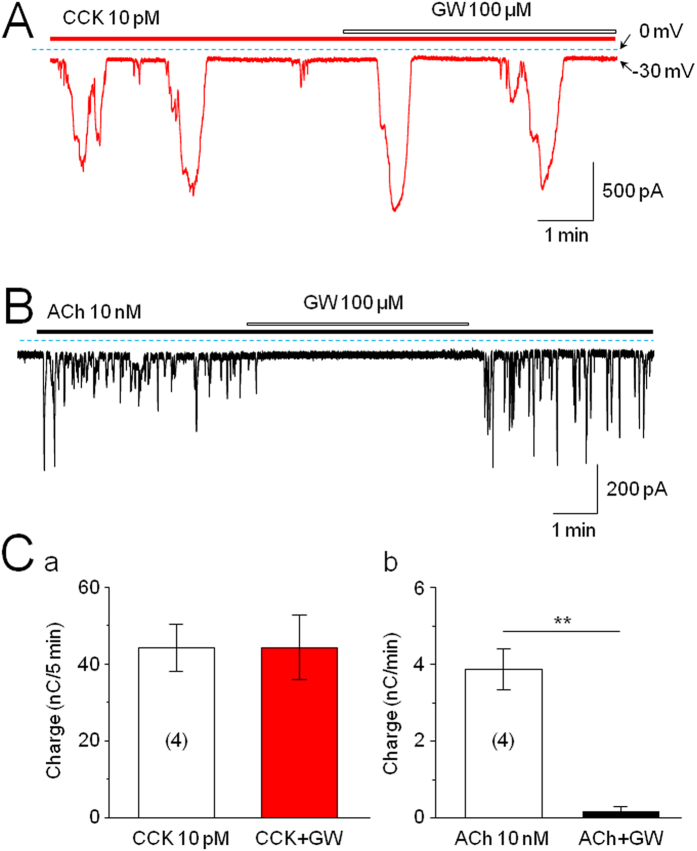
Effects of GW on CCK-induced Ca^2+^ oscillation. (**A**) Representative typical trace of CCK (10 pM)-induced Ca^2+^ oscillations, which are not affected by 100 μM GW. (**B**) In the same recorded cell, ACh (10 nM)-induced Ca^2+^ oscillations are completed eliminated by GW. (**C**) Bar graph summarizes the effect of 100 μM GW on CCK (Ca)- and ACh (Cb)-induced Ca^2+^ oscillations. No asterisk mark (CCK vs CCK + GW 100 μM) means *p* > 0.05 (n = 4). **Indicates *p* < 0.01 compared between ACh 10 nM and ACh + GW 100 μM, n = 4.

Data presented thus far demonstrate that GW inhibited ACh-induced Ca^2+^ oscillations through cell membrane CB_2_Rs, perhaps through CB_2_Rs and muscarinic receptor cross talk. To test this possibility, we applied CCK to induce Ca^2+^ oscillations, which occurs through different receptor signaling pathway than muscarinic receptor, and examined the effects of GW on the CCK-induced Ca^2+^ oscillations. As shown in Fig. 6, bath application of 10 pM CCK induced Ca^2+^ oscillation responses, which were not affected by bath application of GW (100 μM, Fig. 6A). In the same recorded cell, bath application of GW (100 μM) dramatically inhibited 10 nM ACh-induced Ca^2+^ oscillations (Fig. 6B). Figure 6C summarizes pooled data from 4 cells tested, and no significant effect of GW on CCK-induced Ca^2+^ oscillations was found (*p* > 0.05, n = 4, Ca), but GW inhibited ACh-induced Ca^2+^ oscillations in the same recorded cell (*p* < 0.01, n = 4, Cb).

### L-arginine Potentiates ACh-Induced Ca^2+^ Oscillations

**Figure 7:**
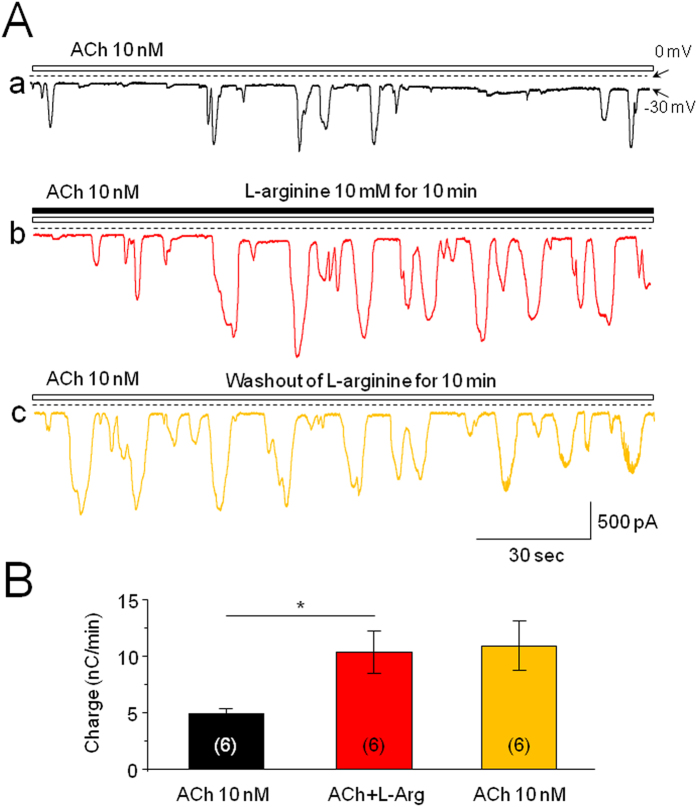
Effects of L-arginine on ACh-induced Ca^2+^ oscillations. (**A**) Representative traces of ACh-induced Ca^2+^ oscillations before (**A**), during (**B**), and after (**C**) bath-application of L-arginine (10 mM, L-Arg). Traces (**A**–**C**) were recorded from the same cell. (**B**) Bar graph summarizes the charge (±SEM) and shows an enhanced effect of L-Arg on ACh-induced Ca^2+^ oscillations. Six cells were assessed before and after L-Arg application. *Indicates *p* < 0.05 compared to baseline level. There was no significance between L-Arg application and washout of L-Arg (p > 0.05), suggesting the effects of L-Arg is non-reversible.

L-arginine is used to induce acute pancreatitis in rodents^22^. In dissociated pancreatic acinar cells, bath-application of L-arginine for 10 min enhanced ACh-induced Ca^2+^ oscillations from baseline level of 4.93 ± 0.39 to 10.34 ± 1.83 nC/min (Fig. 7Aa,b), which was not reversible after washout for 10 min (Ca^2+^ oscillations between L-arginine exposure and washout groups *p* > 0.05, n = 6, Fig. 7Ac). Statistical analysis revealed that L-arginine significantly enhanced ACh-induced Ca^2+^ oscillations (*p* < 0.05) in an irreversible manner (Fig. 7B).

### GW Prevents L-arginine-Enhanced Ca^2+^ Oscillations

**Figure 8:**
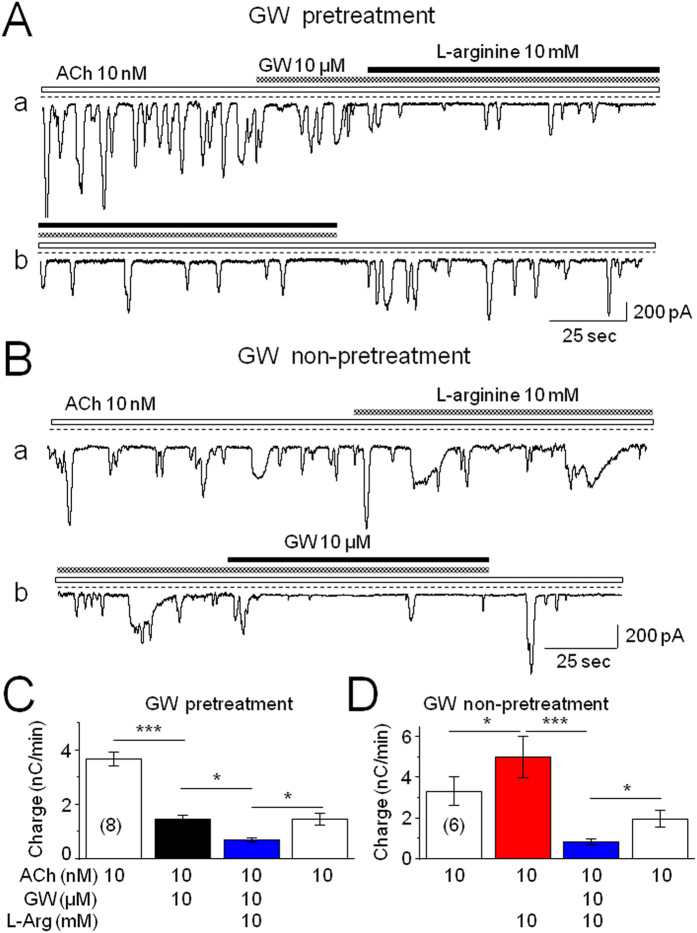
Effects of GW on L-Arg induced enhancement of Ca^2+^ oscillations. (**A**) After pretreatment with GW, bath-applied L-Arg (10 mM for 10 min) fails to enhance ACh-induced Ca^2+^ oscillations. Traces in Fig. 8Aa,b were recorded from the same cell. (**B**) Without pretreatment, bath-applied L-Arg enhances ACh-induced Ca^2+^ oscillations, and under this condition, the addition of GW also reduces L-Arg-induced enhancement of Ca^2+^ oscillations. Traces in Fig. 8Ba,b were recorded from the same cell. (**C**,**D**) GW significantly blocks L-Arg-induced enhancement of Ca^2+^ oscillations either with or without pretreatment of GW. Bar graphs represent averaged charge  ± SEM. The number of cells tested is stated for each condition in parentheses. *Indicates *p* < 0.05, ***Indicates *p* < 0.001.

Next, we sought to determine whether GW could eliminate L-arginine-induced enhancement of Ca^2+^ oscillations. We showed that either pre-treatment with GW (Fig. 8A), or co-administration of GW (10 μM) and L-arginine (Fig. 8B), abolished L-arginine-induced enhancement of Ca^2+^ oscillations (Fig. 8C,D), suggesting that selective activation of acinar cell CB_2_Rs significantly eliminates L-arginine-induced enhancement of intracellular Ca^2+^ signals in mouse pancreatic acinar cells.

### GW Improves L-arginine-Induced Pathology

**Figure 9:**
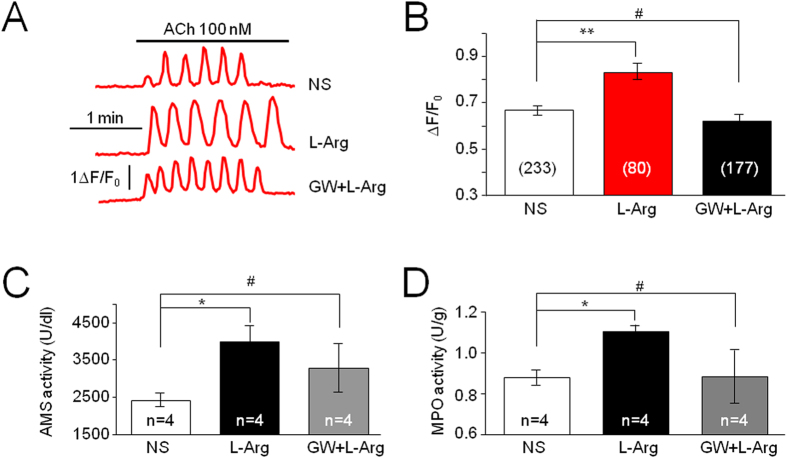
Effects of GW on L-Arg-induced pathology. (**A**) Representative comparison of ACh-induced Ca^2+^ oscillations between the acinar cells prepared from L-Arg (4 g/kg, i.p.) and normal saline (NS) treated mice (after injection for 24 h). These demonstrate an enhancement of ACh-induced Ca^2+^ oscillations in L-Arg-treated mice compared to NS-treated mice. This enhanced effect is prevented by co-injection of GW and L-Arg (GW + L-Arg). (**B**) In these studies, we measured Ca^2+^ responses as ∆F/F_0_, where F refers to the current Fluo signal intensity, F_0_ refers to the background Fluo signal intensity, and ∆F/F_0_ refers to the change of F/F_0_. Using the same measurement, we compared ACh-induced Ca^2+^ responses from pancreatic acinar cells collected from three groups of mice: control (saline-treated mice), L-Arg, and L-Arg plus GW. Compared to the ACh-induced Ca^2+^ oscillations in saline group, there is a significant enhancement of ACh-induced Ca^2+^ oscillations in L-Arg group (**Indicates *p* < 0.01), and there is no statistically significant difference between saline and GW + L-Arg groups (^#^indicates *p* > 0.05), suggesting a prevention of L-Arg-induced enhanced effect by GW. In addition, GW also prevents L-Arg-induced elevation of pancreatic AMS (**C**) and pulmonary MPO (**D**). The number of cells tested is stated for each condition. Bars represent mean ± SEM. In parts C and D, *Indicates *p* < 0.05 between saline and L-Arg groups, but there is no statistically significant difference between saline and L-Arg + GW group (^#^indicates *p* > 0.05).

Finally, we tested whether systemic injection of GW can prevent L-arginine-induced elevation of Ca^2+^ oscillations, and subsequent pathological changes including enhancement of pancreatic amylases (AMS) and pulmonary peritoneal macrophages (MPO) levels, which are two major effects present in early-stage of acute pancreatitis. We injected L-arginine (4.0 g/kg, i.p.) to establish an acute pancreatitis model^23^,^24^, and dissociated pancreatic acinar cells 24 hours later, then compared ACh-induced Ca^2+^ oscillations between saline- and L-arginine-treated groups using Ca^2+^ imaging. Systemic L-arginine injection enhanced ACh-induced Ca^2+^ oscillations compared to systemic saline injection, but GW and L-arginine co-injected showed similar level of ACh-induced Ca^2+^ oscillations (Fig. 9A). Compared to the ACh-induced Ca^2+^ oscillations in saline-treated mice, the acinar cells prepared from L-arginine–treated mice showed a significant increase in Ca^2+^ oscillation response (saline vs. L-arginine group, *p* < 0.01), while co-injection of GW and L-arginine reduced L-arginine’s effect (saline vs. L-arginine + GW group, *p* > 0.05). These results suggest that the activation of pancreatic acinar cell CB_2_Rs may prevent early pathogenesis of acute pancreatitis through the inhibition of enhanced intracellular Ca^2+^ signals. In addition, co-injection of GW (10 mg/kg, i.p.) and L-arginine (4 g/kg, i.p.) also significantly reduced pancreatic L-arginine-induced enhancement of AMS (saline vs. L-arginine, *p* < 0.05, and saline vs. GW + L-arginine, *p* > 0.05; Fig. 9C) and pulmonary MPO levels (saline vs. L-arginine, *p* < 0.05, and saline vs. GW + L-arginine, *p* > 0.05; Fig. 9D). These results suggest that the activation of pancreatic acinar cell CB_2_Rs may prevent early pathogenesis of acute pancreatitis through the inhibition of intracellular Ca^2+^ signals.

## Discussion

The novel findings of this study are that the activation of membrane CB_2_Rs by GW reduces ACh-, but not CCK-induced intracellular Ca^2+^ oscillations, and GW induced reduction of Ca^2+^ oscillations in a concentration-dependent manner. The CB_2_R-mediated reduction of ACh-induced Ca^2+^ oscillations is abolished by pharmacological blockade of CB_2_Rs (AM630) or is absent in CB_2_-KO mice, but not in CB_1_-KO mice. The pancreatitis inducer, L-arginine, significantly enhances ACh-induced intracellular Ca^2+^ oscillations, and the CB_2_R agonist, GW, abolishes this L-arginine effect. In addition, this CB_2_R agonist also improved L-arginine-induced pathological changes. Collectively, our data demonstrate that CB_2_R agonist GW reduces ACh-enhanced intracellular Ca^2+^ signals in mouse pancreatic acinar cells, and this may underlie an important cellular mechanism for a CB_2_R agonist to serve as a new candidate for treating acute pancreatitis.

### CB_2_R Expression in Mouse Pancreatic Acinar Cells

Previously, in rodent pancreatic acinar cells, CB_2_R protein expression was found using immunohistochemical staining and Western blot^10^,^25^. In mouse pancreatic tissue, both CB_1_R and CB_2_R mRNA were identified using real-time RT-PCR and immunohistochemical staining^10^. In the present study, we confirmed that CB_2_R proteins were expressed in freshly isolated mouse pancreatic acinar cells, which is consistent with previous report^10^. Our data demonstrate that CB_2_Rs are expressed in mouse pancreatic acinar cells and they may play an important role in modulating acinar cells function.

### CB_2_R Agonist Reduces ACh-Induced Ca^2+^ Oscillations in Mouse Pancreatic Acinar Cells

Mouse pancreatic acinar cells have been used as an excellent cell model of agonist-induced Ca^2+^ oscillations for studying pancreatitis^26^. We examined whether a selective CB_2_R agonist, GW, affected ACh-induced Ca^2+^ oscillations in the isolated pancreatic acinar cells through CB_2_Rs. Using both patch-clamp recording and confocal Ca^2+^ imaging techniques, we found that GW significantly reduced ACh-induced Ca^2+^ oscillations, and this inhibition is GW-concentration dependent. We also tested another selective CB_2_R agonist, JWH-133, on the ACh-induced Ca^2+^ oscillations, and found a similar inhibition (Supplemental Fig. 4), but the inhibitory effect of JWH-133 was weaker (a higher concentration of JWH-133 was needed compared with GW to induce the same inhibition). It was reported that GW acts as a potent and selective partial agonist for CB_2_R with an EC_50_ of 0.65 nM and selectivity of around 1200× for CB_2_R over CB_1_R^27^,^28^, while JWH-133 has an EC_50_ of 3.4 nM and selectivity of around 200× for CB_2_R over CB_1_R^29^. These findings may explain why GW is more potent than JWH-133 for ACh-induced Ca^2+^ oscillations.

Accumulating evidence demonstrates a complex relationship between the cannabinoid ligand (and receptors) and intracellular Ca^2+^ signals in different types of cells. For example, on one hand, activation of cannabinoid CB_1_R or CB_2_R increased (initiated) intracellular Ca^2+^ levels in endothelia cells^30^, submandibular acinar cells^31^, canine kidney cells^32^, and bladder cancer cells^33^. On the other hand, in pancreatic beta cells, the activation of either CB_1_R^34^ or CB_2_R^35^ reduced glucose-induced intracellular Ca^2+^ oscillations and insulin release. It has been reported that anandamide reduced intracellular Ca^2+^ concentration through the suppression of a Na^+^/Ca^2+^ exchanger current in rat cardiac myocytes^36^. To our knowledge, ours is the first report that a selective CB_2_R agonist reduces intracellular Ca^2+^ signals in mouse pancreatic acinar cells. Considering that Ca^2+^ plays an important role in cellular function, especially enzyme secretion in pancreatic acinar cells, our data suggest that CB_2_R modulates an important aspect of pancreatic acinar cell physiology and pathophysiology.

### CB_2_R Agonist Reduces ACh-Induced Ca^2+^ Oscillations through Membrane CB_2_Rs

Cannabinoid ligands exert their pharmacological effects through CB_1_R or CB_2_R, but in some cases they also can act on non-cannabinoid targets^37^. We determined whether GW modulated intracellular Ca^2+^ signals through a cell membrane or cytosolic CB_2_Rs. First, we examined the effects of pharmacological manipulations of CB_1_R and CB_2_R and found that the CB_2_R selective antagonist AM630 abolished GW-induced reduction of Ca^2+^ oscillations, suggesting that GW modulates ACh-induced Ca^2+^ oscillations through the CB_2_Rs. Then, we genetically manipulated cannabinoid receptors and compared the effects of GW on Ca^2+^ oscillations between WT and CB_2_R-KO mice, and also WT and CB_1_R-KO mice. We found that in CB_2_R-KO but not CB_1_R-KO mice, GW lost its inhibitory effect, further confirming that CB_2_R is the key target for mediating GW-induced reduction in Ca^2+^ oscillations.

In a group of cells tested, we found that a CB_1_R agonist, ACEA (dissolved by ethanol; 10-μM ACEA solution contained 7.3-mM ethanol) reduced ACh-induced Ca^2+^ oscillations (Supplemental Fig. 3); however, the control experiments using the same concentration of ethanol (7.3 mM) also reduced ACh-induced Ca^2+^ oscillations, and the inhibitory effect of ACEA was not absent in the acinar cells dissociated from CB_1_R-KO mice, suggesting a non-specific effect, likely caused by ethanol. In addition, we also tested the effects of DMSO (GW was dissolved by DMSO to 100 mM stock solution), and found that 1 μM DMSO itself did not affect ACh-induced Ca^2+^ oscillations (Supplemental Fig. 5). Together, our data support the conclusion that GW selectively acts on acinar cell CB_2_Rs and reduces ACh-induced Ca^2+^ oscillations.

Finally, we asked where the CB_2_Rs are located (membrane or cytosolic CB_2_Rs). To address this question, we designed three experiments. We first examined the effect of bath-applied GW on the Ca^2+^ oscillations induced by intracellular application of IP_3_, and found that GW did not affect IP_3_-induced Ca^2+^ oscillations, suggesting that the target that mediated GW-induced inhibition in Ca^2+^ oscillations is located in the signal pathway before IP_3_ receptors, and not on the IP_3_ receptor itself. We then intracellularly applied GW through a recording electrode to examine the effect of intracellular administration of GW on bath ACh-induced Ca^2+^ oscillations, and found that intracellular infusion of GW (even at 100 μM) did not alter ACh-induced Ca^2+^ oscillations. Finally, we intracellularly applied AM630 through a recording electrode to examine the effect of bath-applied GW on ACh-induced Ca^2+^ oscillations. Our data showed that intracellular infusion of AM630 did not prevent bath-applied GW-induced reduction of Ca^2+^ oscillations. Collectively, our data support the conclusion that GW modulates intracellular Ca^2+^ signaling through the membrane CB_2_Rs in pancreatic acinar cells.

### Possible Mechanisms of GW-Induced Reduction in ACh-Induced Ca^2+^ Oscillations

The precise mechanism by which GW modulates intracellular Ca^2+^ signals is unclear. Our data show that membrane CB_2_Rs are necessary for mediating GW’s effect. GW’s action in ACh-induced Ca^2+^ oscillations should occur at the G-protein-mediated signal pathway between muscarinic receptor (M_3_) activation and IP_3_ production because GW did not affect IP_3_-induced Ca^2+^ oscillations. We also demonstrated that GW failed to affect ACh-induced Ca^2+^ oscillations in pancreatic acinar cells prepared from CB_2_R-KO mice, suggesting that GW likely did not affect muscarinic receptor function. In addition, we found that bath-applied GW failed to inhibit CCK-induced Ca^2+^ oscillations even at 100 μM, suggesting that GW selectively modulates muscarinic receptor-mediated G-protein signaling^38^. Therefore, the possible mechanisms for GW-induced modulation of ACh-induced Ca^2+^ oscillations may involve cross talk between muscarinic receptor- and CB_2_R-mediated G-protein signal pathways, such as homologous and/or heterologous desensitization of G-protein coupled receptors (GPCRs)^39^. For example, in the case of homologous desensitization of GPCRs, the activation of one type of GPCR can rapidly terminate another GPCR signaling through the internalization of receptors after binding, phosphorylation of G-protein coupled receptor kinases, and formation of complexes with β-arresting^39^,^40^. In addition, the activation of a GPCR may also result in temporary inhibition of another GPCR signal through a heterologous desensitization, which does not involve receptor internalization, but activation of several signal transduction pathways, particularly protein kinase C (PKC)- and PKA-dependent signaling pathways^38^,^41^. It has been reported that intracellular cyclic AMP-generated substances play an important role in regulation of IP_3_ and Ca^2+^ responses to ACh in rat submandibular acini. Investigators found that intracellular cAMP increased IP_3_ formation in response to ACh, while blocking PKA by H89 reduced IP_3_ formation^41^. Because it is well known that the activation of CB_2_Rs significantly reduces intracellular cAMP levels, we thus postulated that GW may activate CB_2_Rs, reduce cAMP, and in turn reduce intracellular IP_3_ production, and lead to a reduction of ACh-induced Ca^2+^ oscillations. Our findings warrant further testing of this hypothesis.

### Clinical Significance of CB_2_R-Mediated Reduction of Ca^2+^ Oscillations in Pancreatic Acinar Cells

Pancreatic acinar cells are functional units of the exocrine pancreas. They synthesize, store, and secrete inactive preforms of digestive enzymes into the lumen of the acinus. The activity of pancreatic acinar cells is crucially modulated by the secretagogues ACh and CCK; both can act on their specific membrane receptors (muscarinic and CCK receptor, respectively) and then induce an elevation in cytoplasmic calcium. If high concentrations of intracellular Ca^2+^ persist, intracellular signaling is disrupted, cell damage occurs, and acute pancreatitis forms. Emerging evidence suggests that the earliest abnormalities of acute pancreatitis arise by aberrant elevation of intracellular Ca^2+^ within acinar cells because the sustained intracellular Ca^2+^ elevation activates intracellular digestive proenzymes resulting in necrosis and inflammation, and pharmacological blockade of store-operated or Ca^2+^ release-activated Ca^2+^ channels would prevent sustained elevation of intracellular Ca^2+^, and consequence protease activation and necrosis^3^. In the present study, we provide the first evidence that the CB_2_R agonist, GW, reduces ACh-induced Ca^2+^ oscillations, abolishes L-arginine–induced enhancement of Ca^2+^ oscillations and prevents L-arginine–induced elevation of both pancreatic AMS and pulmonary MPO levels. These results suggest that a CB_2_R agonist may serve as a novel therapeutic strategy to prevent and/or treat acute pancreatitis. This conclusion is consistent with previous report that a CB_2_R agonist exhibits a protective effect on pathogenesis in an acute pancreatitis animal model^10^. Our data showing a reduction of intracellular Ca^2+^ signaling by GW also provide a new target to interpret the role of CB_2_R agonists in treating acute pancreatitis in addition to CB_2_R-mediated anti-inflammation.

## Additional Information

**How to cite this article**: Huang, Z. *et al*. Cannabinoid receptor subtype 2 (CB_2_R) agonist, GW405833 reduces agonist-induced Ca^2+^ oscillations in mouse pancreatic acinar cells. *Sci. Rep.* **6**, 29757; doi: 10.1038/srep29757 (2016).

## Supplementary Material

Supplementary Information

## References

[b1] Pandol S. J., Saluja A. K., Imrie C. W. & Banks P. A. Acute pancreatitis: bench to the bedside. Gastroenterology 132, 1127–1151 (2007). 17383433 10.1053/j.gastro.2007.01.055

[b2] Bakker O. J. . Treatment options for acute pancreatitis. Nature reviews. Gastroenterology & hepatology 11, 462–469 (2014). 24662281 10.1038/nrgastro.2014.39

[b3] Gerasimenko J. V. . Ca2+ release-activated Ca2+ channel blockade as a potential tool in antipancreatitis therapy. Proceedings of the National Academy of Sciences of the United States of America 110, 13186–13191 (2013). 23878235 10.1073/pnas.1300910110 PMC3740877

[b4] Munro S., Thomas K. L. & Abu-Shaar M. Molecular characterization of a peripheral receptor for cannabinoids. Nature 365, 61–65 (1993). 7689702 10.1038/365061a0

[b5] Kaminski N. E. Immune regulation by cannabinoid compounds through the inhibition of the cyclic AMP signaling cascade and altered gene expression. Biochemical pharmacology 52, 1133–1140 (1996). 8937419 10.1016/0006-2952(96)00480-7

[b6] Herring A. C., Koh W. S. & Kaminski N. E. Inhibition of the cyclic AMP signaling cascade and nuclear factor binding to CRE and kappaB elements by cannabinol, a minimally CNS-active cannabinoid. Biochemical pharmacology 55, 1013–1023 (1998). 9605425 10.1016/s0006-2952(97)00630-8

[b7] Basu S., Ray A. & Dittel B. N. Cannabinoid receptor 2 is critical for the homing and retention of marginal zone B lineage cells and for efficient T-independent immune responses. Journal of immunology 187, 5720–5732 (2011). 10.4049/jimmunol.1102195 PMC3226756 22048769

[b8] Cheng Y. & Hitchcock S. A. Targeting cannabinoid agonists for inflammatory and neuropathic pain. Expert opinion on investigational drugs 16, 951–965 (2007). 17594182 10.1517/13543784.16.7.951

[b9] Pertwee R. G. The diverse CB1 and CB2 receptor pharmacology of three plant cannabinoids: delta9-tetrahydrocannabinol, cannabidiol and delta9-tetrahydrocannabivarin. British journal of pharmacology 153, 199–215 (2008). 17828291 10.1038/sj.bjp.0707442 PMC2219532

[b10] Michler T. . Activation of cannabinoid receptor 2 reduces inflammation in acute experimental pancreatitis via intra-acinar activation of p38 and MK2-dependent mechanisms. American journal of physiology. Gastrointestinal and liver physiology 304, G181–G192 (2013). 23139224 10.1152/ajpgi.00133.2012

[b11] Zimmer A., Zimmer A. M., Hohmann A. G., Herkenham M. & Bonner T. I. Increased mortality, hypoactivity, and hypoalgesia in cannabinoid CB1 receptor knockout mice. Proceedings of the National Academy of Sciences of the United States of America 96, 5780–5785 (1999). 10318961 10.1073/pnas.96.10.5780 PMC21937

[b12] Buckley N. E. . Immunomodulation by cannabinoids is absent in mice deficient for the cannabinoid CB(2) receptor. European journal of pharmacology 396, 141–149 (2000). 10822068 10.1016/s0014-2999(00)00211-9

[b13] Wu J. . Thimerosal modulates the agonist-specific cytosolic Ca2+ oscillatory patterns in single pancreatic acinar cells of mouse. FEBS Lett 390, 149–152 (1996). 8706847 10.1016/0014-5793(96)00646-1

[b14] Wu J. . 2-Aminoethoxydiphenyl borate modulates kinetics of intracellular Ca(2+) signals mediated by inositol 1,4,5-trisphosphate-sensitive Ca(2+) stores in single pancreatic acinar cells of mouse. Mol Pharmacol 58, 1368–1374 (2000). 11093775 10.1124/mol.58.6.1368

[b15] Huang Z. B. . Congo red modulates ACh-induced Ca(2+) oscillations in single pancreatic acinar cells of mice. Acta pharmacologica Sinica 35, 1514–1520 (2014). 25345744 10.1038/aps.2014.94 PMC4261129

[b16] Yang K. . Distinctive nicotinic acetylcholine receptor functional phenotypes of rat ventral tegmental area dopaminergic neurons. J Physiol 587, 345–361 (2009). 19047205 10.1113/jphysiol.2008.162743 PMC2670049

[b17] Wu J. . 2-aminoethoxydiphenyl borate inhibits agonist-induced Ca2+ signals by blocking inositol trisphosphate formation in acutely dissociated mouse pancreatic acinar cells. Pflugers Archiv: European journal of physiology 448, 592–595 (2004). 15197598 10.1007/s00424-004-1295-0

[b18] Wakui M., Potter B. V. & Petersen O. H. Pulsatile intracellular calcium release does not depend on fluctuations in inositol trisphosphate concentration. Nature 339, 317–320 (1989). 2498663 10.1038/339317a0

[b19] Wakui M., Osipchuk Y. V. & Petersen O. H. Receptor-activated cytoplasmic Ca2+ spiking mediated by inositol trisphosphate is due to Ca2(+)-induced Ca2+ release. Cell 63, 1025–1032 (1990). 1701691 10.1016/0092-8674(90)90505-9

[b20] Osipchuk Y. V., Wakui M., Yule D. I., Gallacher D. V. & Petersen O. H. Cytoplasmic Ca2+ oscillations evoked by receptor stimulation, G-protein activation, internal application of inositol trisphosphate or Ca2+: simultaneous microfluorimetry and Ca2+ dependent Cl- current recording in single pancreatic acinar cells. The EMBO journal 9, 697–704 (1990). 1690123 10.1002/j.1460-2075.1990.tb08162.x PMC551723

[b21] den Boon F. S. . Excitability of prefrontal cortical pyramidal neurons is modulated by activation of intracellular type-2 cannabinoid receptors. Proceedings of the National Academy of Sciences of the United States of America 109, 3534–3539 (2012). 22331871 10.1073/pnas.1118167109 PMC3295302

[b22] Zhang J. & Rouse R. L. Histopathology and pathogenesis of caerulein-, duct ligation-, and arginine-induced acute pancreatitis in Sprague-Dawley rats and C57BL6 mice. Histology and histopathology 29, 1135–1152 (2014). 24585404 10.14670/HH-29.1135

[b23] Kui B. . New insights into the methodology of L-arginine-induced acute pancreatitis. PloS one 10, e0117588 (2015). 25688985 10.1371/journal.pone.0117588 PMC4331527

[b24] Yu L. T. . Recombinant Reg3alpha protein protects against experimental acute pancreatitis in mice. Molecular and cellular endocrinology 422, 150–159 (2016). 26683606 10.1016/j.mce.2015.12.002

[b25] Linari G. . Involvement of cannabinoid CB1- and CB2-receptors in the modulation of exocrine pancreatic secretion. Pharmacological research: the official journal of the Italian Pharmacological Society 59, 207–214 (2009). 10.1016/j.phrs.2008.11.002 19070664

[b26] Petersen O.H. Ca^2+^ signaling in pancreatic acinar cells: physiology and pathophysiology. Braz J Med Biol Res 42, 9–16 (2009). 19219293 10.1590/s0100-879x2009000100003

[b27] Marriott K. S. & Huffman J. W. Recent advances in the development of selective ligands for the cannabinoid CB(2) receptor. Current topics in medicinal chemistry 8, 187–204 (2008). 18289088 10.2174/156802608783498014

[b28] Huffman J. W. The search for selective ligands for the CB2 receptor. Current pharmaceutical design 6, 1323–1337 (2000). 10903395 10.2174/1381612003399347

[b29] Huffman J. W. . 3-(1′,1′-Dimethylbutyl)-1-deoxy-delta8-THC and related compounds: synthesis of selective ligands for the CB2 receptor. Bioorganic & medicinal chemistry 7, 2905–2914 (1999). 10658595 10.1016/s0968-0896(99)00219-9

[b30] Zoratti C., Kipmen-Korgun D., Osibow K., Malli R. & Graier W. F. Anandamide initiates Ca(2+) signaling via CB2 receptor linked to phospholipase C in calf pulmonary endothelial cells. Br J Pharmacol 140, 1351–1362 (2003). 14645143 10.1038/sj.bjp.0705529 PMC1574152

[b31] Kopach O. . Cannabinoid receptors in submandibular acinar cells: functional coupling between saliva fluid and electrolytes secretion and Ca2+ signalling. Journal of cell science 125, 1884–1895 (2012). 22366450 10.1242/jcs.088930

[b32] Chou K. J. . CP55,940 increases intracellular Ca2+ levels in Madin-Darby canine kidney cells. Life sciences 69, 1541–1548 (2001). 11554615 10.1016/s0024-3205(01)01242-5

[b33] Jan C. R. . Novel effect of CP55,940, a CB1/CB2 cannabinoid receptor agonist, on intracellular free Ca2+ levels in bladder cancer cells. The Chinese journal of physiology 45, 33–39 (2002). 12005350

[b34] Nakata M. & Yada T. Cannabinoids inhibit insulin secretion and cytosolic Ca2+ oscillation in islet beta-cells via CB1 receptors. Regulatory peptides 145, 49–53 (2008). 17884194 10.1016/j.regpep.2007.08.009

[b35] Juan-Pico P. . Cannabinoid receptors regulate Ca(2+) signals and insulin secretion in pancreatic beta-cell. Cell calcium 39, 155–162 (2006). 16321437 10.1016/j.ceca.2005.10.005

[b36] Li Q., Cui N., Du Y., Ma H. & Zhang Y. Anandamide reduces intracellular Ca2+ concentration through suppression of Na+/Ca2+ exchanger current in rat cardiac myocytes. PloS one 8, e63386 (2013). 23667607 10.1371/journal.pone.0063386 PMC3646750

[b37] Xiong W. . Cannabinoids suppress inflammatory and neuropathic pain by targeting alpha3 glycine receptors. The Journal of experimental medicine 209, 1121–1134 (2012). 22585736 10.1084/jem.20120242 PMC3371734

[b38] Doi R., Chowdhury P. & Rayford P. L. Agonist-regulated alteration of the affinity of pancreatic muscarinic cholinergic receptors. The Journal of biological chemistry 268, 22436–22443 (1993). 7693670

[b39] Mohle R. & Drost A. C. G protein-coupled receptor crosstalk and signaling in hematopoietic stem and progenitor cells. Annals of the New York Academy of Sciences 1266, 63–67 (2012). 22901257 10.1111/j.1749-6632.2012.06559.x

[b40] Luttrell L. M. & Lefkowitz R. J. The role of beta-arrestins in the termination and transduction of G-protein-coupled receptor signals. Journal of cell science 115, 455–465 (2002). 11861753 10.1242/jcs.115.3.455

[b41] Martinez J. R. & Zhang G. H. Cross-talk in signal transduction pathways of rat submandibular acinar cells. European journal of morphology 36 Suppl, 190–193 (1998). 9825920

