# Supplementary material for: Cannabinoid receptor subtype 2 (CB2R) agonist, GW405833 reduces agonist-induced Ca2+ oscillations in mouse pancreatic acinar cells
Source: Sci Rep. 2016 Jul 19;6:29757. doi: 10.1038/srep29757 (PMC4949433; doi:10.1038/srep29757)
Supplement: Supplementary Information [file srep29757-s1.doc]

#### Cannabinoid receptor subtype 2 (CB_2_R) agonist, GW405833 reduces agonist-induced Ca^2+^ oscillations in mouse pancreatic acinar cells

Zebing Huang^1,2^, Haiyan Wang^3^, Jingke Wang^3#^, Mengqin Zhao^3^, Nana Sun^3^, Fangfang Sun^3^, Jianxin Shen^3^, Haiying Zhang^4^, Kunkun Xia^2#^, Dejie Chen^2#^, Ming Gao^2^, Ronald P. Hammer Jr^5,6^, Qingrong Liu^4^, Zhengxiong Xi^4^, Xuegong Fan^1*^, Jie Wu^1,2,3,5*^

^1^Department of Infectious Diseases, Xiangya Hospital, Central South University, and Key Laboratory of Viral Hepatitis, Hunan Province, Changsha 410008, China

^2^Departments of Neurology and Neurobiology, Barrow Neurological Institute, St. Joseph’s Hospital and Medical Center, Phoenix AZ 85013, USA

^3^Department of Physiology, Shantou University Medical College, Shantou, Guangdong 515041, China

^4^Intramural Research Program, National Institute on Drug Abuse, Baltimore, MD 21224, USA

^5^Department of Basic Medical Sciences, University of Arizona College of Medicine, Phoenix, AZ 85004, USA

**Correspondence:** Jie Wu, MD, PhD

Professor

Divisions of Neurology and Neurobiology

Barrow Neurological Institute

St. Joseph’s Hospital and Medical Center

350 W. Thomas Rd.; Phoenix, AZ 85013

Voice: (602) 406-6376; Fax: (602) 406-7172

E-mail: jie.wu@dignityhealth.org

^*^Xuegong Fan and Jie Wu play equal roles as corresponding authors for his paper.

^#^Jingke Wang’s recent address: Department of Laboratory Medicine, Lishui People's Hospital, Lishui, Zhejiang 323000, China

^#^Kunkun Xia’s recent address: ^1^Department of General Surgery, The First Affiliated Hospital of Zhengzhou University, Zhengzhou, Henan, 450052, China

^#^Dejie Chen’s recent address: Department of Neurology, Yun-Fu People’s Hospital, Yun-Fu, Guangdong, 527300, China


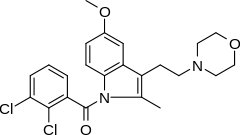


**Supplemental Figure 1.** Chemical structure of the GW405833 (GW)


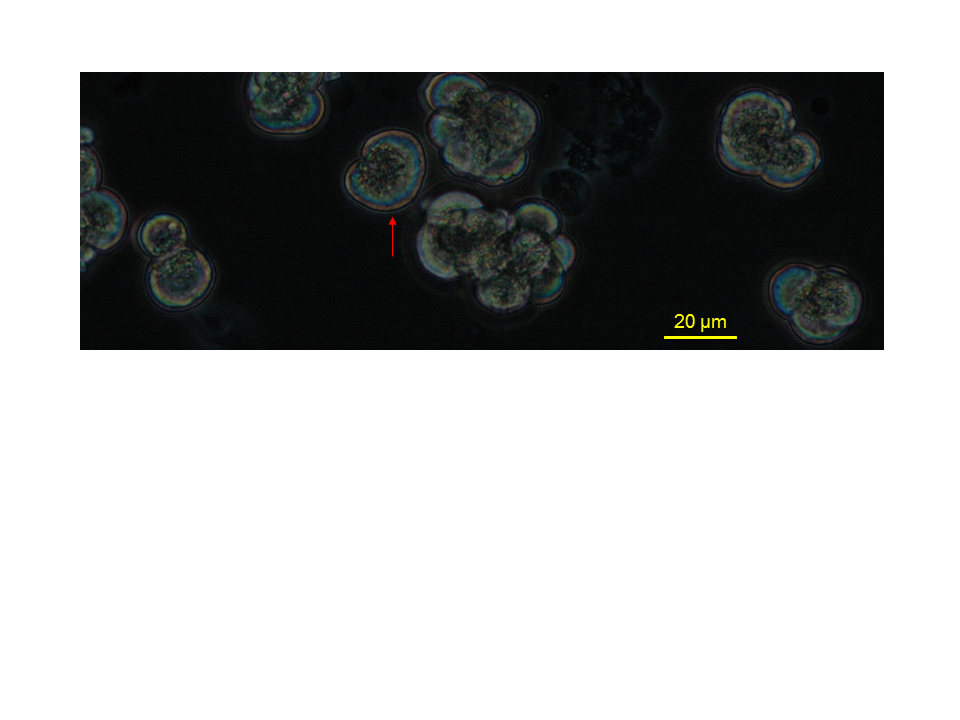


**Supplemental Figure 2.** A phase-photo picture of an acutely dissociated mouse pancreatic acinar cell, demonstrating a typical kidney shape with the secretion granules in the central area.


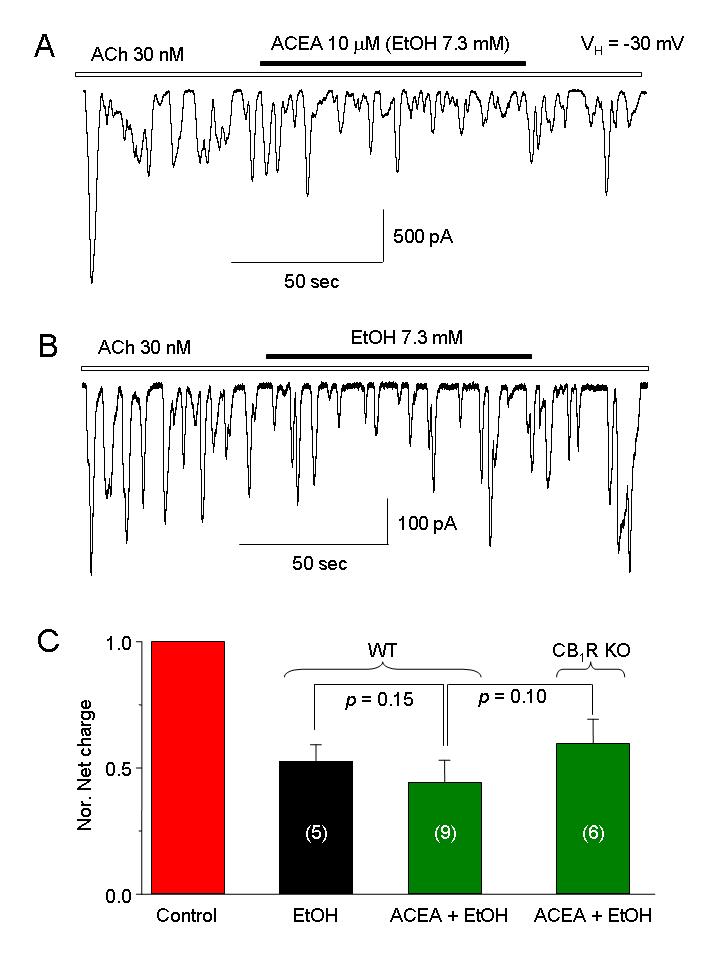


**Supplemental Figure 3.** Effects of the CB_1_R agonist (ACEA, 10 μM) on ACh-induced Ca^2+^ oscillations. **(A)** A typical trace of the effects of ACEA (containing 7.3-mM ethanol) on ACh-induced Ca^2+^ oscillations. **(B)** Effects of control (7.3-mM ethanol) on ACh-induced Ca^2+^ oscillations. **(C)** Statistical analysis showed similar effects of ACEA and 7.3-mM ethanol on ACh-induced Ca^2+^ oscillations (*p*=0.15). Importantly, the ACEA-induced reduction in Ca^2+^ oscillations was still present in the pancreatic acinar cells dissociated from the CB_1_R-KO mice, suggesting that CB_1_Rs do not participate in the modulation in ACh-induced Ca^2+^ oscillations.


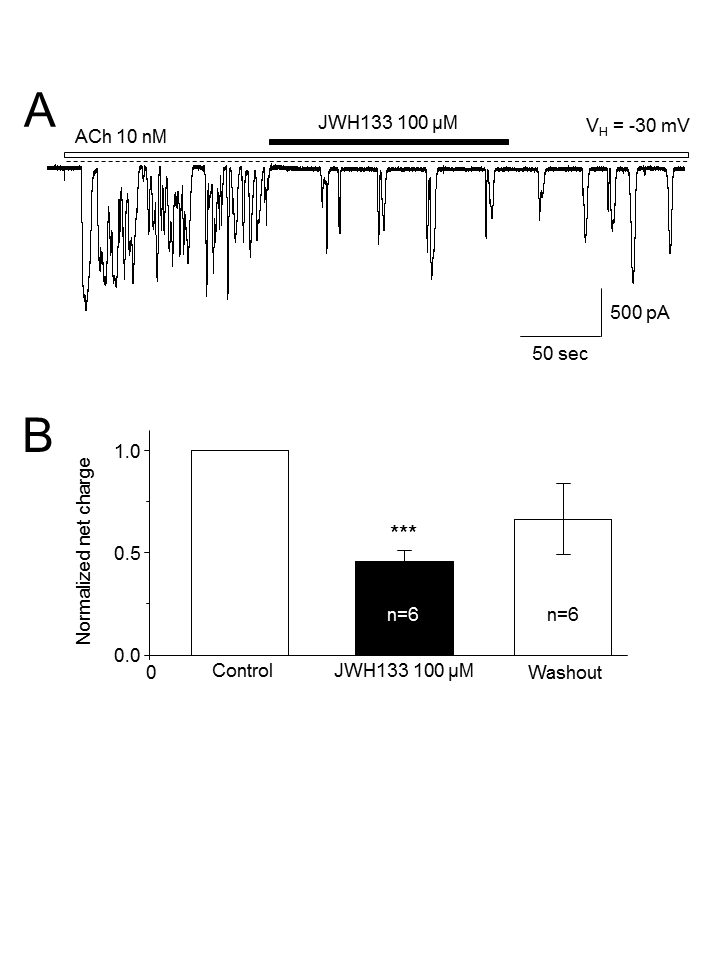


**Supplemental Figure 4.** Effects of the CB_2_R agonist, JWH-133, on ACh-induced Ca^2+^ oscillations. **(A)** A typical trace of the effect of JWH-133 (100 µM) on 10-nM ACh-induced Ca^2+^ oscillations. **(B)** The pooled data show the inhibition of ACh-induced Ca^2+^ oscillations by JWH-133. *** indicates *p*<0.001.


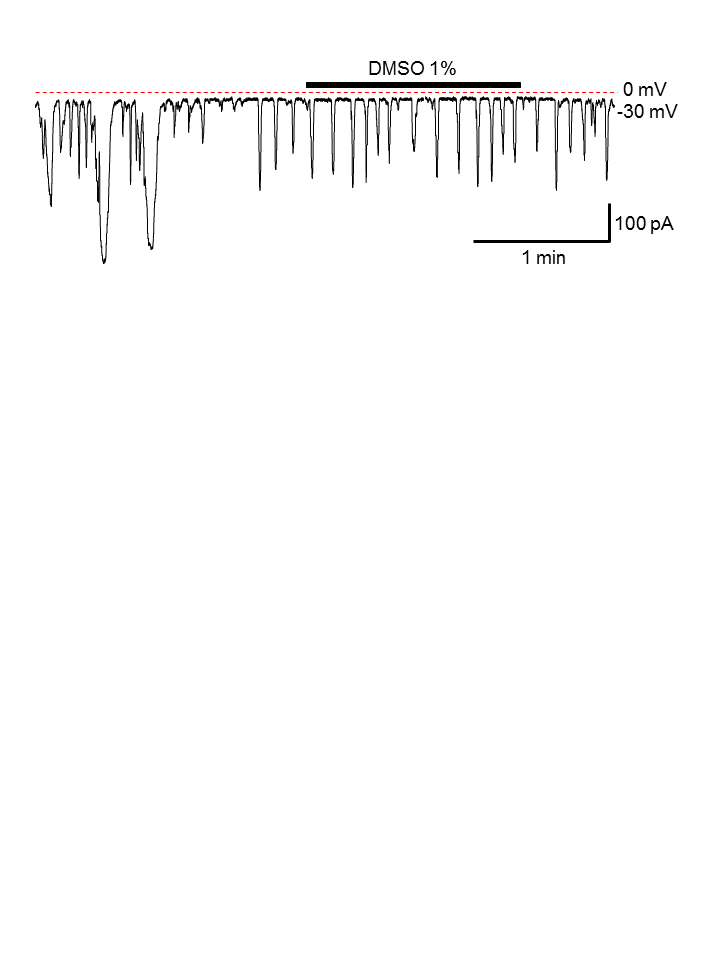


**Supplemental Figure 5.** Effects of DMSO (1 µM) on ACh-induced Ca^2+^ oscillations. A representative trace result indicating that 1-µM DMSO did not affect ACh-induced Ca^2+^ oscillations in acutely dissociated mouse pancreatic acinar cells. Similar results were observed in another three cell tests.
